# Complement Factor C5a Is Increased in Blood of Patients with Abdominal Aortic Aneurysm and Has Prognostic Potential for Aneurysm Growth

**DOI:** 10.1007/s12265-020-10086-5

**Published:** 2020-12-17

**Authors:** Branislav Zagrapan, Wolf Eilenberg, Andreas Scheuba, Johannes Klopf, Annika Brandau, Julia Story, Katharina Dosch, Hubert Hayden, Christoph M. Domenig, Lukas Fuchs, Rüdiger Schernthaner, Robin Ristl, Ihor Huk, Christoph Neumayer, Christine Brostjan

**Affiliations:** 1 Department of Surgery: Division of Vascular Surgery and Surgical Research Laboratories, Medical University of Vienna, Vienna General Hospital, Vienna, Austria; 2 Department of Biomedical Imaging and Image Guided Therapy: Division of Cardiovascular and Interventional Radiology, Medical University of Vienna, Vienna General Hospital, Vienna, Austria; 3 Center for Medical Statistics, Informatics, and Intelligent Systems, Medical University of Vienna, Vienna, Austria

**Keywords:** Abdominal aortic aneurysm, C3a, C5a, Diagnosis, Leukotriene B4, Prognosis

## Abstract

In this observational case-control study, circulating levels of complement factors C3a and C5a and leukotriene B4 (LTB4) were analysed in abdominal aortic aneurysm (AAA) patients regarding their association with diagnosis and prognosis. Serum C5a was significantly raised in AAA patients compared to healthy controls—median 84.5 ng/ml (IQR = 37.5 ng/ml) vs. 67.7 ng/ml (IQR = 26.2 ng/ml), *p* = 0.007—but was not elevated in patients with athero-occlusive disease. Serum C5a levels correlated significantly with the increase in maximum AAA diameter over the following 6 months (*r* = 0.319, *p* = 0.021). The median growth in the lowest quartile of C5a (< 70 ng/ml) was 50% less compared to the highest C5a quartile (> 101 ng/ml): 1.0 mm/6 months (IQR = 0.8 mm) vs. 2.0 mm/6 months (IQR = 1.5 mm), *p* = 0.014. A log-linear mixed model predicted AAA expansion based on current diameter and C5a level. To our knowledge, this is the first study linking complement activation, in particular C5a serum level, with AAA progression.

**Graphical Abstract:**

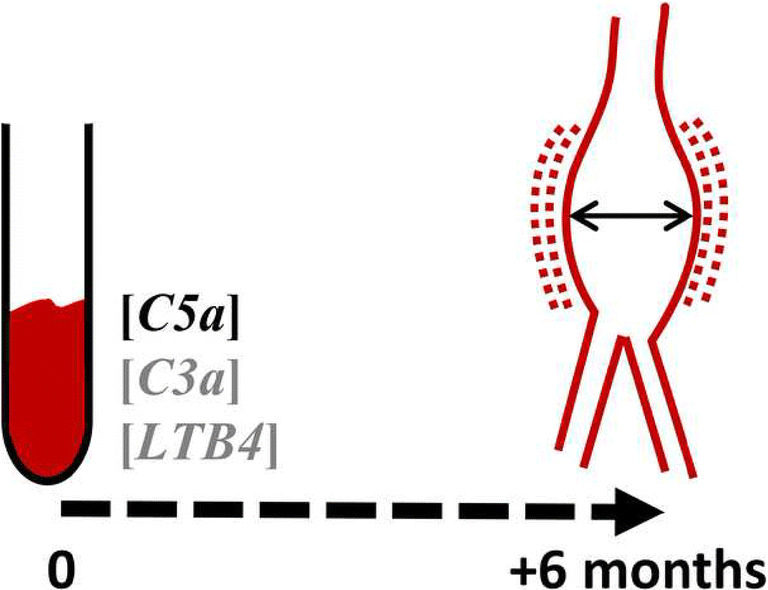

**Supplementary Information:**

The online version contains supplementary material available at 10.1007/s12265-020-10086-5.

## Introduction

Abdominal aortic aneurysm (AAA) is a degenerative disease, caused by weakening and dilatation of the abdominal aortic wall [1]. It affects primarily elderly Caucasian men whose overall risk for AAA development as well as risk of AAA progression is strongly associated with direct cigarette smoke exposure [2]. Sterile inflammation is a significant component of AAA pathogenesis with characteristic leukocyte and macrophage infiltration in AAA wall media and adventitia [3]. The intraluminal thrombus (ILT), which is frequently found at the AAA site, seems to be biomechanically relevant by reducing peak wall stress [4] but is also biologically active [5]. In addition to adventitial inflammation, the ILT may contribute to the enhanced degradation of the aortic wall [6]. Leukocytes, erythrocytes and platelets accumulating mainly in the luminal ILT layer release oxidative and proteolytic molecules which promote AAA wall destruction. Furthermore, they secrete chemotactic mediators which may increase the number and types of cells recruited [6]. Of note, local inflammatory and haemostatic activity at the AAA site seems to be echoed at the systemic level as measured by several cellular and humoral factors in peripheral blood [7, 8]. These components were proposed as markers of disease activity and potentially predict the rate of progression or risk of rupture [9].

The recruitment of neutrophils to the AAA site is driven by interleukin 8 (IL-8) and leukotriene B4 (LTB4) which may be produced by neutrophils themselves from platelet-released arachidonic acid [10, 11]. Knock-out or drug treatment of the LTB4 receptor significantly reduced aneurysm formation in experimental AAA models [12, 13]. Histological examination and in vitro migration assays revealed neutrophil-derived LTB4 as a major chemotactic factor released from the ILT [14]. To date, no comprehensive analysis of LTB4 blood levels in AAA patients has been conducted.

The complement system has also been shown to be associated with AAA pathogenesis and neutrophil chemotaxis. Elements of the complement system have been found in all layers of human AAA tissue [15]. Functionally, both C3a and C5a have shown potent chemoattractant activity for neutrophils in a murine model of AAA and the concomitant blockade of C3a and C5a activity prevented aneurysm formation [15]. Antibody-mediated responses against matrix proteins in the degenerating aortic wall have been proposed as a trigger of complement activation in AAA [16, 17]. On a systemic level, increased circulating C3a and C5a have been linked to progressive vascular disease such as increased risk of cardiovascular events [18], restenosis after superficial femoral artery balloon angioplasty [19] and advanced aortic calcification in dialysis patients [20]. In AAA patients, plasma levels of C3 rather than C3a or C5a have previously been investigated but yielded contradictory results [21, 22].

The present study relates to the finding that neutrophil recruitment and engagement is essential in AAA development as reflected in reduced aneurysm formation upon neutrophil depletion in mice [23]. Since several chemoattractants for granulocytes have been detected at the aneurysm site [24], we aimed to compare the circulating levels of C3a, C5a and LTB4 in AAA patients and investigate their association with aneurysm growth.

## Materials and Methods

### Study Design

The presented human studies were approved by the Vienna General Hospital Ethics Committee (no 1729/2014) adhering to the principles in the Declaration of Helsinki. All patients and healthy volunteers gave informed consent for participation in the study.

An observational case-control study was conducted to investigate the marker potential of C3a, C5a and LTB4 in a diagnostic setting, including 37 healthy individuals and 40 AAA patients recruited sequentially at the vascular surgery outpatient clinic of the Vienna General Hospital with known or newly diagnosed AAA. The groups were matched with respect to age, body mass index (BMI) and smoker status. A subgroup analysis was performed for 33 healthy individuals and 33 AAA patients without peripheral arterial occlusive disease (PAD) who were compared to 24 PAD patients. Control subjects (healthy individuals and PAD patients) were recruited among general surgery, vascular surgery, ophthalmology and urology patients in whom AAA absence was confirmed by ultrasound. Exclusion criteria for all groups were recent tumour or chemotherapy (< 1 year), autoimmune disease, haematological disease, instable angina pectoris and organ transplantation. Blood samples were collected from all participants. Concomitantly, computed tomographic angiography (CTA) was performed for AAA patients.

In the prognostic setting, an exploratory cohort of 28 AAA patients was investigated between October 2014 and June 2018, adhering to the same exclusion criteria. Morphometric analysis of AAA was performed using CTA images at the time of study inclusion and at 6-month intervals thereafter. Corresponding with CTA imaging, peripheral venous blood was collected for analysis of blood-borne factors.

In addition to the exploratory cohort (where monitoring time points were restricted to 1–2 follow-up visits, i.e. within a time frame of 6–12 months), an extended data set of an ongoing long-term screening study (2014–2020) was investigated including 66 AAA patients with up to 4 years of follow-up at 6-month intervals.

### Image Acquisition and Analysis

The CT angiography of the abdominal aorta was acquired using our institutional standard protocol. Until 2017, all CT angiographies were performed on a Siemens Flash device (Siemens Healthineers, Forchheim, Germany). Starting from 2017, a Siemens Force (Siemens Healthineers) was used. The acquired data was analysed using the CT vascular module of syngo.via (Siemens Healthineers). The software automatically identifies the centre line of the aorta in order to provide well-aligned orthogonal views of the aortic cross-section. The maximum AAA diameter (AAA dmax) was measured with semi-automatic tools provided within the framework, with intra- and interobserver variability ranging at 0.14 mm and 0.20 mm, respectively. The measurements were rounded to within the nearest 0.1 mm.

### Analysis of Blood Parameters

Peripheral venous blood was collected into serum tubes with clotting activator (Greiner Bio One, Kremsmünster, Austria), was kept at room temperature for 1–2 h, and then centrifuged at 1000 x g for 10 min at room temperature and aliquots were stored at − 80 °C until analysis. C5a and C3a levels in serum were measured using commercial ELISAs with antibodies against C5a-desArg and C3a-desArg as per manufacturer’s instructions (BD Biosciences, San Diego, CA, USA). LTB4 was assessed with a competitive ELISA by Bio-Techne (Minneapolis, MN, USA) in plasma prepared from blood samples anticoagulated with citrate, theophylline, adenosine and dipyridamole and centrifuged twice (1000 x g, 10,000 x g) for 10 min at 4 °C. For the purposes of this study, a set of clinical haematological and biochemical parameters available from the Vienna General Hospital laboratory service was evaluated.

### Statistical Evaluation

The results are presented as median and interquartile range (IQR) for continuous variables and counts with percent sample group for nominal variables. For assessment of statistical significance, non-parametric tests were employed (Mann-Whitney *U* test for group comparisons of continuous variables, Spearman test for correlations, chi-square or Fisher exact test for nominal variables). The diagnostic marker potential was characterized by receiver operating characteristic (ROC) analysis. Multivariable binary logistic regression was conducted to assess the association of serum C5a with presence of AAA adjusting for comorbidities and medication. In the prognostic setting, slow versus fast disease progression was defined as < 2- or ≥ 2-mm increase in AAA diameter over 6 months matching previously published cut-offs at 4 mm per year [25]. To analyse the association of C5a level and AAA growth rate with longitudinal monitoring data, a specifically tailored log-linear mixed model was employed. In detail, the natural logarithm of the AAA dmax ratio between two subsequent measurements was explained through the time interval between the two measurements (in years) and the interaction of time interval and C5a serum level as measured at the beginning of the time interval. The effect of time interval was modelled as normally distributed random effect at patient level to account for correlations of repeated observations within the same patient. C5a levels were centred at the sample mean before entering the model to allow for direct estimation of the average growth rate. No intercept was included, because at a theoretical time interval of length zero, the AAA growth is zero by definition. Baseline dmax is not included as predictor, because the model explains relative change, and hence, the initial dmax of each time interval is included in the log-transformed dmax ratio which is the outcome variable of the model. Two-sided *p* values below 0.05 were considered as statistically significant. Due to the explorative character of the study, no adjustment for multiple testing was applied. The data analysis was performed with SPSS (Version 25.0, SPSS Inc., Chicago, IL, USA).

## Results

### C5a Serum Level Is Increased in AAA

Forty patients, 3 women and 37 men, with advanced AAA were included in this study. Thirty-seven subjects, 5 women and 32 men, comprised the control group. The median age was 70.6 years in the AAA group (IQR = 10.7) and 68.0 years (IQR = 14.3) in the control group (Table 1). Median BMI was 27.9 (IQR = 5.6) and 26.5 (IQR = 5.0) in the AAA and control groups, respectively. In both collectives, most patients were current (52.5% of AAA and 29.7% of healthy individuals) or ex-smokers (37.5% in AAA and 45.9% in the healthy group). Median AAA dmax was 57.2 mm (IQR = 11.9 mm) and median maximum ILT thickness ranged at 21.6 mm (IQR = 10.5 mm). While matched in sex, age, BMI and smoker status, patients and controls differed significantly in risk factors and comorbidities associated with AAA such as hypertension, hyperlipidaemia, coronary heart disease and chronic obstructive pulmonary disease as well as in the pertaining medication (Table 2).

**Table 1:** Demographics of AAA patients and controls in the diagnostic study

Parameter		Healthy (*N* = 37)		AAA (*N* = 40)		*p* value
		Median	IQR	Median	IQR	
Age (years)		68.0	14.3	70.6	10.7	0.838
Body mass index		26.5	5.0	27.9	5.6	0.668
Nicotine pack-years		25.0	34.5	40.0	32.5	0.072
Maximum AAA diameter (mm)				57.2	11.9	
Aneurysm volume (cm^3^)				145.3	84.3	
Maximum ILT thickness (mm)				21.6	10.5	
		N	%	N	%	
Sex	Women	5	13.5	3	7.5	0.470
	Men	32	86.5	37	92.5	
Smoker	Never	9	24.3	4	10.0	0.080
	Past	17	45.9	15	37.5	
	Current	11	29.7	21	52.5	
AAA family history	Yes	4	10.8	4	10.0	0.624
	No	33	89.2	35	87.5	
	Unknown	0	0.0	1	2.5	

*AAA* abdominal aortic aneurysm, *ILT* intraluminal thrombus, *IQR* interquartile range

**Table 2:** Comorbidities and medication of AAA patients and controls in the diagnostic study

Comorbidity	Healthy (*N* = 37)	AAA (*N* = 40)	*p* value
	*N* (%)	*N* (%)	
Hypertension	23 (62.2%)	34 (85.0%)	0.022
Hyperlipidaemia	11 (29.7%)	33 (82.5%)	< 0.001
PAD	4 (10.8%)	7 (17.5%)	0.402
Coronary heart disease	4 (10.8%)	14 (35.0%)	0.016
Myocardial infarction	3 (8.1%)	9 (22.5%)	0.082
Stroke	3 (8.1%)	2 (5.0%)	0.667
Diabetes mellitus	4 (10.8%)	9 (22.5%)	0.171
COPD	4 (10.8%)	14 (35.0%)	0.012
Anti-platelet therapy	12 (32.4%)	37 (92.5%)	< 0.001
Anti-coagulation therapy	6 (16.2%)	9 (22.5%)	0.487
Anti-hypertensive therapy	23 (62.2%)	34 (85.0%)	0.022
Lipid-lowering agents	6 (16.2%)	34 (85.0%)	< 0.001
Diabetic medication	4 (10.8%)	7 (17.5%)	0.402
	Median (IQR)	Median (IQR)	
Framingham risk score [%]	23.6 (24.3)	30.4 (25.7)	0.031

*AAA* abdominal aortic aneurysm, *COPD* chronic obstructive pulmonary disease, *IQR* interquartile range, *PAD* peripheral artery occlusive disease

**Fig. 1:**
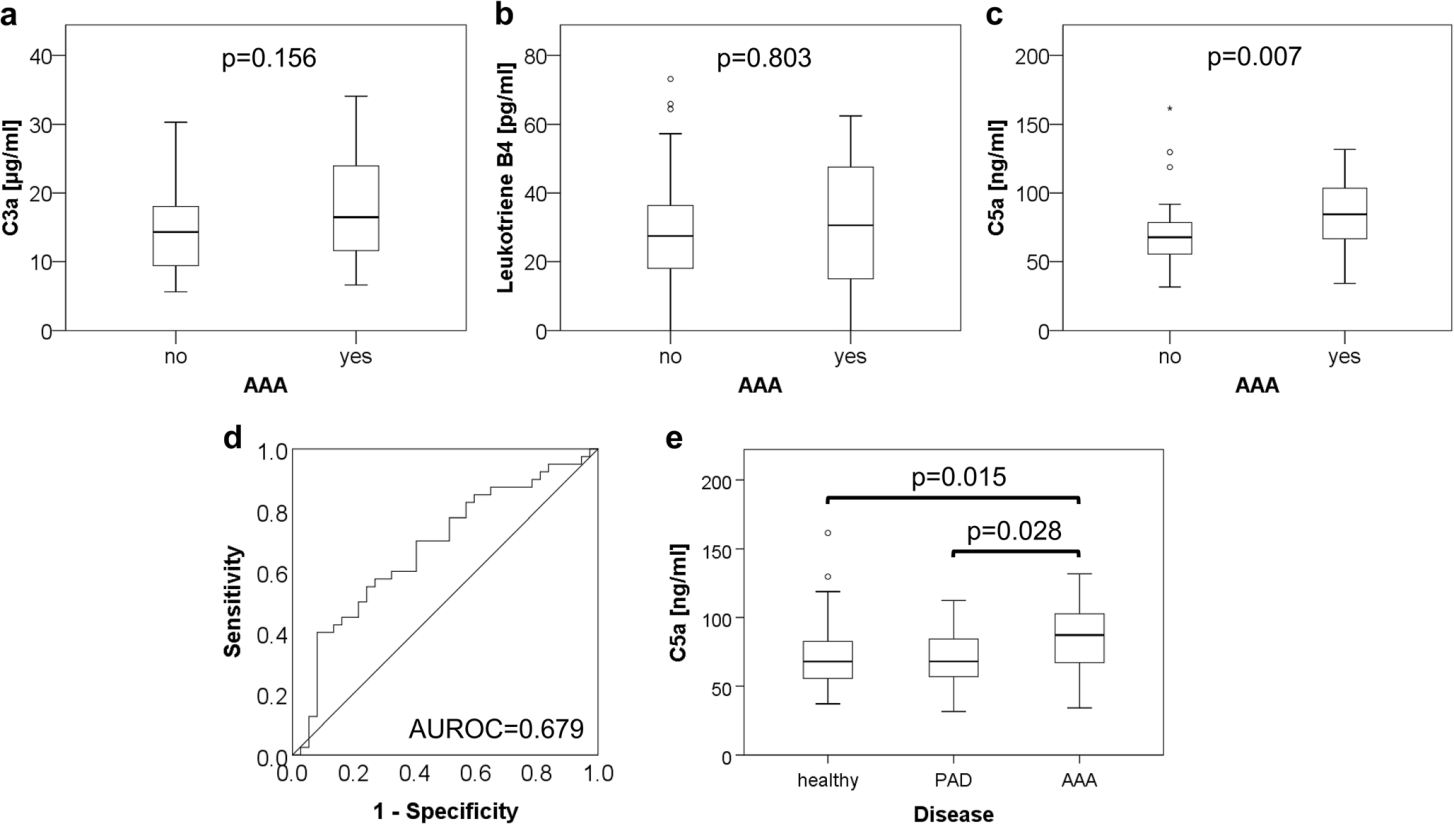
Comparison of circulating C3a, C5a and LTB4 between AAA patients and controls. The complement factors C3a and C5a were measured in serum samples, while leukotriene B4 was assessed in plasma collected from AAA (*N* = 40) and healthy controls (*N* = 37). Boxplots illustrate the distribution of circulating levels of **a** C3a, **b** LTB4 and **c** C5a; *p* values refer to group comparisons by Mann-Whitney *U* test. **d** The diagnostic marker value of C5a was further evaluated by receiver operating characteristic (ROC) analysis and the area under the ROC curve (AUROC). **e** Serum C5a levels were further compared between healthy controls (*N* = 33) and patients with peripheral arterial disease (PAD, *N* = 24) or AAA (N = 33) by Mann-Whitney *U* test

The explorative blood parameters C3a and LTB4 showed a moderate but statistically non-significant elevation of median levels among the AAA patient group compared to the healthy control group with C3a: 16.5 μg/ml (IQR = 12.5 μg/ml) vs. 14.3 μg/ml (IQR = 9.1 μg/ml), *p* = 0.156 and LTB4: 30.6 pg/ml (IQR = 33.6 pg/ml) vs. 27.5 pg/ml (IQR = 18.8 pg/ml), *p* = 0.803 (Fig. 1a, b). On the contrary, circulating C5a was found to be significantly raised in AAA patients compared to controls: 84.5 ng/ml (IQR = 37.5 ng/ml) vs. 67.7 ng/ml (IQR = 26.2 ng/ml), *p* = 0.007 (Fig. 1c). Using ROC analysis to test the association of C5a with AAA disease state, an area under the ROC curve (AUROC) of 0.679 was obtained (Fig. 1d). A moderate correlation was found between serum C5a and C3a levels (*r* = 0.339, *p* = 0.003). LTB4 did not correlate significantly with either C5a or C3a. Regarding potential confounders, circulating C5a levels were further compared between individuals with and without hypertension, hyperlipidaemia, coronary heart disease and COPD and were found to be significantly higher in the COPD group (Suppl. Table 1). Yet, multivariable regression analysis identified serum C5a, hyperlipidaemia, anti-platelet therapy and lipid-lowering agents as independent parameters associated with AAA disease state (Suppl. Tables 2 and 3). Further support of the notion that serum C5a is an independent biomarker not affected by hyperlipidaemia or atherosclerosis was gained from a comparison of patients (*N* = 24) with peripheral arterial occlusive disease (PAD; Suppl. Table 4). In a subgroup analysis that included the 33 AAA patients and 33 healthy controls without PAD (Fig. 1e), circulating C5a was significantly elevated in AAA but not in PAD patients as compared to healthy individuals: median (IQR) ranging at 87.2 (36.9), 67.9 (28.0) and 67.9 (31.0) ng/ml C5a, respectively.

### C5a Is Associated with AAA Progression

Next, we were interested whether the serum C5a level at the start of a 6-month period was associated with an increase in AAA dmax recorded at the end of this period. In an exploration cohort of 28 AAA patients (Table 3), fifty-two such 6-month monitoring periods of the C5a level and the corresponding AAA morphometric measurements were performed. The median time between the C5a serum measurements was 6.1 months (IQR = 0.9 months, range 4.7–9.7 months). The corresponding median AAA measurement interval by the CT scan was 6.2 months (IQR = 1.2, range 4.1–9.3 months). The majority of these patients were male (*n* = 23, 82%), with a median age of 71.5 years (IQR = 9.4) and a history of smoking (*n* = 26, 93%). While the AAA dmax at baseline ranged at 50.8 mm (IQR = 10.4), the patients showed a median AAA dmax increase of 1.25 mm over 6 months (IQR = 1.5, range 0.1–6.4 mm).

**Table 3:** Patient demographics in explorative prognostic study at baseline

Parameter		*N* (%)	Median (IQR)
Sex	Women	5 (17.9%)	
	Men	23 (82.1%)	
Age (years)			71.5 (9.4)
Body mass index			27.7 (7.2)
Smoker	Never	2 (7.1%)	
	Past	17 (60.7%)	
	Current	9 (32.1%)	
Nicotine pack-years			34.0 (27.0)
AAA family history		2 (7.1%)	
Maximum AAA diameter (mm)			50.8 (10.4)
Aneurysm volume (cm^3^)			99.9 (60.6)
Maximum ILT thickness (mm)			17.1 (16.9)
Hypertension		24 (85.7%)	
Hyperlipidaemia		26 (92.9%)	
PAD		7 (25.0%)	
Coronary heart disease		12 (42.9%)	
Myocardial infarction		7 (25.0%)	
Stroke		4 (14.3%)	
Diabetes mellitus		9 (32.1%)	
COPD		8 (28.6%)	
Anti-platelet therapy		26 (92.9%)	
Anti-hypertensive therapy		23 (82.1%)	
Lipid-lowering agents		24 (85.7%)	

*AAA* abdominal aortic aneurysm, *COPD* chronic obstructive pulmonary disease, *ILT* intraluminal thrombus, *IQR* interquartile range, *PAD* peripheral artery occlusive disease

**Fig. 2:**
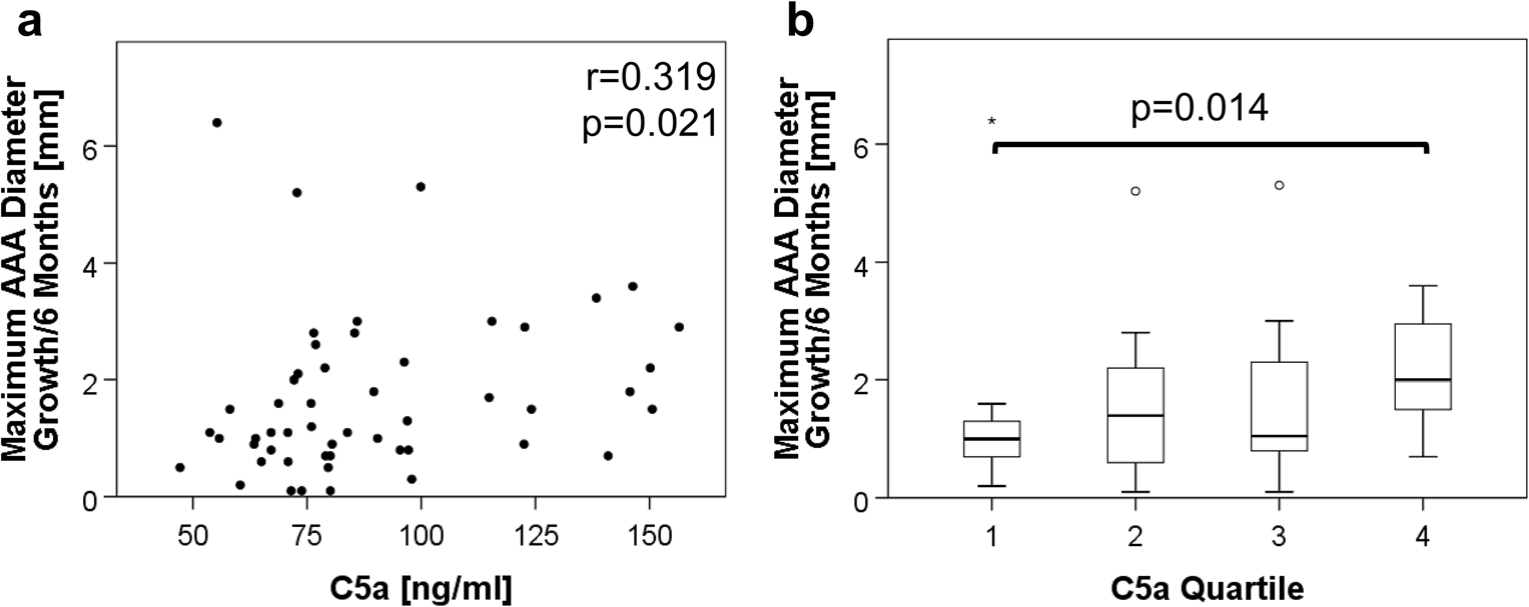
Association between baseline serum C5a and AAA growth in the following 6 months. For the exploration set of fifty-two 6-month monitoring periods, **a** the correlation of C5a serum values with subsequent AAA growth (in mm of maximal aortic diameter) over the next 6 months is illustrated by scatter plot and Spearman correlation coefficient. **b** AAA growth rates are depicted by boxplots according to C5a quartiles

Notably, the serum C5a level correlated significantly with the increase in AAA dmax over the following 6 months (r = 0.319, *p* = 0.021, Fig. 2a). The patients in the lowest C5a quartile showed a significantly slower median growth in AAA dmax of 1.0 mm/6 months (IQR = 0.8 mm) compared to patients in the highest C5a quartile displaying a median AAA enlargement of 2.0 mm/6 months (IQR = 1.5, *p* = 0.014, Fig. 2b). Patients with a C5a level above the 75th percentile (>101 ng/ml) had an odds ratio for rapid expansion (≥2 mm/6 months) of 11 (95% CI 1.1–114.1) compared with those under the 25th percentile (<70 ng/ml). Slow and rapid progressors did not differ significantly in the investigated demographic variables (Suppl. Table 5). However, when C5a serum levels, AAA baseline diameter as well as major comorbidities and medication were included in multivariable analysis (Suppl. Table 6), C5a, hypertension and diabetes mellitus prevailed as independent prognostic indicators.

Since this exploration set was limited to only 28 patients who had completed the surveillance study with mostly 1–2 monitoring periods, we further extended the marker evaluation by a preliminary analysis of ongoing, long-term screening patients (Suppl. Table 7). A total of 66 patients with repeated blood and CTA analyses (*N* = 153) over a follow-up range of 6–48 months was included. While a significant correlation of C5a levels with AAA dmax (*r* = 0.187, *p* = 0.020, *N* = 153) as well as a significant difference in AAA expansion over the next 6 months was retained for patients with C5a serum levels in the lowest versus highest C5a quartile (*p* = 0.039), the inclusion of longer observation periods introduced a considerable bias of multiple 6-month data sets originating from the same individual. Specifically, the calculation of significance levels did not account for inter-dependence of measurements. Thus, statistical analysis was performed with a log-linear mixed model (incorporating time as the main effect and C5a serum level as effect modificator) which confirmed the significance of serum C5a to predict AAA growth (Table 4). The model-based equation of y_t_ = y_0_ * e^(lambda * time + beta * (biomarker value at baseline y0 – mean) * time^ would thus project an AAA dmax y_t_ of 41.8 mm versus 42.6 mm within a year’s time for a patient with a starting diameter y_0_ of 40 mm and a C5a serum level in the lowest (60 ng/ml) or highest (120 ng/ml) quartile, respectively (mean C5a value: 83 ng/ml).

**Table 4:** Log-linear mixed model for AAA growth prediction: estimates of fixed effects

Parameter	Estimate	95% CI Lower value	95% CI upper value	*p* value
CTA interval (years)	0.050826 (lambda)	0.041291	0.060362	< 0.001
Interaction of CTA interval and baseline C5a level	0.000309 (beta)	0.000022	0.000569	0.035

## Discussion

The focus of this study was the evaluation of three neutrophil chemotaxins for their association with AAA and disease progression. Of the evaluated factors C3a, C5a and LTB4, only circulating C5a differed significantly between AAA patients and healthy controls. Moreover, C5a was not elevated in patients with athero-occlusive disease and was found to be associated with the increase in AAA maximum diameter in the 6-month period following the C5a measurement.

These three chemotaxins were specifically chosen as they have previously been shown to mediate neutrophil recruitment to abdominal aortic aneurysms [24, 26] and represent signals which are amplified by activated neutrophils. While C3a and LTB4 showed a trend for elevated median serum levels in AAA patients, the differences to control subjects were statistically insignificant, possibly due to the high variability within groups. Thus, despite their local accumulation at the aneurysm site [15, 24], the release of C3a and LTB4 into systemic circulation does not seem to be a sensitive measure of AAA development. In contrast, circulating serum C5a could discriminate between AAA patients and healthy individuals at a statistically significant level (AUROC = 0.679, *p* = 0.007). Considering that all three factors investigated in this study are also known to be elevated in cardiovascular disease other than AAA [27–30], it could be hypothesised that complement activation was more prominent in the AAA patients who showed more extensive underlying atherosclerotic disease than the healthy controls, reflected by a Framingham risk score of 30% (AAA patients) versus 24% (control subjects), respectively (*p* = 0.031, Table 2). Yet, serum C5a and hyperlipidaemia were identified as independent parameters associated with AAA disease state in multivariable analysis. Furthermore, in a comparison of patients with peripheral arterial occlusive disease, circulating C5a was significantly elevated in AAA patients while healthy individuals and PAD controls did not differ. Thus, serum C5a exhibits discriminatory power between AAA and atherosclerotic disease.

While the intraluminal thrombus associated with abdominal aortic aneurysms has previously been reported as a site of complement activation and hence a source of increased circulating complement components [15], we did not detect a correlation between the serum level of C5a and maximum ILT thickness or the increase in maximum ILT thickness over the 6-month follow-up period (data not shown). A possible explanation is that ILT thickness may not be directly related to its biological activity. On the one hand, a thicker thrombus is presumed to increase the AAA wall inflammatory activity via effecting local AAA wall hypoxia [31]. On the other hand, a thinner ILT has shown higher proteolytic activity (e.g. higher levels of matrix metalloproteinase 9, MMP-9) and accumulation of neutrophils in the luminal layer physically closer to the AAA wall [32, 33]. Yet, when we compared MMP-9 plasma levels and C5a serum levels in the aneurysm patients, no correlation was found (data not shown). These findings indicate that elevated circulating C5a might not only originate from the ILT but also from the other main inflammatory AAA site, the adventitia.

Notably, in an explorative prognostic setting, we could identify a correlation between serum C5a levels and the AAA maximum diameter measured 6 months later. The median increase in AAA dmax in patients with C5a level above the 75th percentile was double compared to patients with C5a below the 25th percentile. Moreover, a C5a serum level above the 75th percentile was associated with an 11-fold odds ratio of rapid expansion (≥ 2 mm/6 months). The independent prognostic value of serum C5a was confirmed in multivariable analysis (in addition to a positive association of hypertension and negative association of diabetes mellitus with aneurysm expansion, as previously recognized [34]). Unexpectedly, the AAA baseline diameter did not differ significantly between slow and fast progressors. This finding might be explained by the circumstance that 50% of AAA dmax baseline measurements in this cohort were between 48 and 57 mm, i.e. within a rather narrow size range. Also, C5a serum levels did not correlate with baseline diameter but with AAA growth rate which may indicate that this parameter reflects a more active, inflammatory disease state rather than current aortic expansion.

When extending the C5a analysis to a preliminary data set from an ongoing monitoring study, a log-linear mixed model accounting for multiple measurements in patients over time confirmed the predictive potential of serum C5a for aneurysm growth.

To our knowledge, this is the first study linking complement activation and C5a with AAA progression. While the results have been acquired from a limited patient sample, they are exploratory in nature and highlight the interesting association between C5a and AAA risk of progression. In particular, the presented log-linear mixed model would offer the possibility to make individual predictions of aneurysm expansion based not only on current diameter but also on a blood parameter influenced by the inflammatory AAA state.

## Supplementary Information

ESM 1 (PDF 96.7 kb)

## Abbreviations

AAA: Abdominal aortic aneurysm
AAA dmax: Maximum diameter of the abdominal aortic aneurysm
AUC: Area under the curve
AUROC: Area under the ROC curve
BMI: Body mass index
C3a: Complement factor C3a
C5a: Complement factor C5a
COPD: Chronic obstructive pulmonary disease
ILT: Intraluminal thrombus
IQR: Interquartile range
LTB4: Leukotriene B4
MMP-9: Matrix metalloproteinase 9
PAD: Peripheral arterial occlusive disease
ROC: Receiver operating characteristic
