# Supplementary material for: Complement Factor C5a Is Increased in Blood of Patients with Abdominal Aortic Aneurysm and Has Prognostic Potential for Aneurysm Growth
Source: J Cardiovasc Transl Res. 2020 Dec 17;14(4):761–9. doi: 10.1007/s12265-020-10086-5 (PMC8397625; doi:10.1007/s12265-020-10086-5)
Supplement: Supplementary file 1 — (PDF 96.7 kb) [file 12265_2020_10086_MOESM1_ESM.pdf]

SUPPLEMENTAL DATA

Complement Factor C5a is Increased in Blood of Patients with Abdominal Aortic Aneurysm and has Prognostic Potential for Aneurysm Growth

Branislav Zagrapan, M.D.[a], Wolf Eilenberg, M.D., Ph.D.[a], Andreas Scheuba[a], Johannes Klopf, BSc, M.D.[a], Annika Brandau[a], Julia Story[a], Katharina Dosch, M.D.[a], Hubert Hayden, MSc.[a], Christoph M. Domenig, M.D.[a], Lukas Fuchs[a], Rüdiger Schernthaner, M.D.[b], Robin Ristl, Ph.D.[c], Ihor Huk, M.D.[a], Christoph Neumayer, M.D.[a], Christine Brostjan, Ph.D.[a,*]

Medical University of Vienna, Vienna General Hospital, Vienna, Austria:

[a]Department of Surgery: Division of Vascular Surgery and Surgical Research Laboratories

[b]Department of Biomedical Imaging and Image Guided Therapy: Division of Cardiovascular and Interventional Radiology

[c]Center for Medical Statistics, Informatics, and Intelligent Systems

* Corresponding author: Prof. Christine Brostjan, Ph.D.

Department of Surgery, Medical University of Vienna

Anna Spiegel Centre for Translational Research

Vienna General Hospital 25.05.002, A-1090 Vienna, Austria

Phone: +43 1 40400 73514

Fax: +43 1 40400 73593

Email: christine.brostjan@meduniwien.ac.at

SUPPLEMENTAL TABLES

Supplemental Table 1 Serum values of C5a according to co-morbidities in the diagnostic study

Co-morbidity (Frequency)	No	Yes	p-value
C5a [ng/ml]	*Median (IQR)*	*Median (IQR)*	
Hypertension (74%)	72.6 (23.6)	75.8 (42.0)	0.944
Hyperlipidaemia (57%)	74.9 (33.5)	71.3 (40.1)	0.885
Coronary heart disease (23%)	74.9 (38.7)	70.1 (37.0)	0.434
COPD (23%)	71.1 (34.5)	83.3 (36.0)	0.044

COPD, chronic obstructive pulmonary disease; IQR, interquartile range.

Supplemental Table 2 Multivariable analysis (binary logistic regression) of AAA diagnosis including major risk factors and comorbidities

Parameter	Exp(B)	95% CI Lower value	95% CI Upper value	p-value
Serum C5a [ng/ml]	1.031	1.006	1.057	0.015
Smoking	2.630	0.437	15.826	0.291
Hypertension	1.846	0.441	7.729	0.402
Hyperlipidaemia	12.313	3.126	48.507	<0.001
Coronary heart disease	1.915	0.376	9.753	0.434
COPD	2.882	0.579	14.355	0.196
Constant	0.005			0.001

CI, confidence interval; COPD, chronic obstructive pulmonary disease; Exp(B), odds ratio.

Supplemental Table 3 Multivariable analysis (binary logistic regression, method Enter) of AAA diagnosis including medication*

Parameter	Exp(B)	95% CI Lower value	95% CI Upper value	p-value
Serum C5a [ng/ml]	1.041	1.010	1.072	0.009
Anti-platelet therapy	17.158	2.634	111.751	0.003
Anti-hypertensive therapy	1.009	0.142	7.149	0.993
Lipid-lowering agents	19.217	4.039	91.435	<0.001
Constant	0.002			<0.001

CI, confidence interval; Exp(B), odds ratio.

*Please note that due to collinearity between comorbidities and medication (hypertension/anti-hypertensive therapy and hyperlipidaemia/lipid-lowering agents) two separate multivariable analyses are conducted.

Supplemental Table 4 Demographics of AAA versus PAD patients

Parameter		PAD (N=24)		AAA (N=33)		p-value
		Median	IQR	Median	IQR	
Age [years]		74	13	71	11	0.053
Body-mass index		25.6	3.4	28.1	6.2	0.219
Nicotine pack-years		37.0	61.5	40.0	37.0	0.769
Maximum AAA diameter [mm]				56.3	12.6	
Aneurysm volume [cm^3]				136.4	90.3	
Maximum ILT thickness [mm]				21.6	9.9	
		**N**	**%**	**N**	**%**	
Sex	Women	0	0.0%	3	9.1%	0.256
	Men	24	100.0%	30	90.9%	
Smoking	Yes	21	87.5%	29	87.9%	1.000
	No	3	12.5%	4	12.1%	
Hypertension	Yes	22	91.7%	27	81.8%	0.446
	No	2	8.3%	6	18.2%	
Hyperlipidaemia	Yes	17	70.8%	26	78.8%	0.491
	No	7	29.2%	7	21.2%	
Coronary heart disease	Yes	12	50.0%	12	36.4%	0.303
	No	12	50.0%	21	63.6%	
Diabetes mellitus	Yes	9	37.5%	9	27.3%	0.412
	No	15	62.5%	24	72.7%	
COPD	Yes	8	33.3%	11	33.3%	1.000
	No	16	66.7%	22	66.7%	
Anti-platelet therapy	Yes	19	79.2%	30	90.9%	0.261
	No	5	20.8%	3	9.1%	
Anti-hypertensive therapy	Yes	20	83.3%	27	81.8%	1.000
	No	4	16.7%	6	18.2%	
Lipid-lowering agents	Yes	19	79.2%	27	81.8%	1.000
	No	5	20.8%	6	18.2%	

AAA, abdominal aortic aneurysm; COPD, chronic obstructive pulmonary disease; ILT, intraluminal thrombus; IQR, interquartile range; PAD, peripheral artery occlusive disease.

Supplemental Table 5 Demographics of AAA patients with slow (<2 mm/6 months) or rapid aneurysm expansion (≥2 mm/6 months)

Parameter		Slow progression periods (N=35)		Fast progression periods (N=17)		p-value
		**Median**	**IQR**	**Median**	**IQR**	
Age [years]		74.3	9.5	71.3	11.2	0.441
Body-mass index		27.4	8.2	27.4	7.6	0.423
Nicotine pack-years		30.0	39.8	30.0	30.0	0.818
Maximum AAA diameter [mm]		50.9	10.6	52.1	8.8	0.513
Aneurysm volume [cm^3]		104.8	66.8	119.0	74.0	0.092
Maximum ILT thickness [mm]		16.9	12.5	17.8	13.5	0.816
		**N**	**%**	**N**	**%**	
Sex	Women	4	11.4	5	29.4	0.133
	Men	31	88.6	12	70.6	
Smoker	Yes	32	91.4	15	88.2	1.000
	No	3	8.6	2	11.8	
Hypertension	Yes	34	97.1	14	82.4	0.097
	No	1	2.9	3	17.6	
Hyperlipidaemia	Yes	35	100.0	15	88.2	0.103
	No	0	0.0	2	11.8	
PAD	Yes	9	25.7	2	11.8	0.304
	No	26	74.3	15	88.2	
Coronary heart disease	Yes	15	42.9	6	35.3	0.602
	No	20	57.1	11	64.7	
Diabetes mellitus	Yes	10	28.6	8	47.1	0.189
	No	25	71.4	9	52.9	
COPD	Yes	5	14.3	4	23.5	0.451
	No	30	85.7	13	76.5	
Anti-platelet therapy	Yes	33	94.3	16	94.1	1.000
	No	2	5.7	1	5.9	
Anti-hypertensive therapy	Yes	31	88.6	13	76.5	0.413
	No	4	11.4	4	23.5	
Lipid-lowering agents	Yes	32	91.4	13	76.5	0.198
	No	3	8.6	4	23.5	

AAA, abdominal aortic aneurysm; COPD, chronic obstructive pulmonary disease; ILT, intraluminal thrombus; IQR, interquartile range; PAD, peripheral artery occlusive disease.

Supplemental Table 6 Multivariable analysis (binary logistic regression) of AAA prognosis including AAA dmax and all parameters with p<0.200 as listed in Suppl. Table 5

**Parameter**	**Exp(B)**	**95% CI Lower value**	**95% CI Upper value**	**p-value**
Serum C5a [ng/ml]	1.038	1.003	1.073	0.031
Maximum AAA diameter [mm]	1.025	0.866	1.214	0.774
Aneurysm volume [cm^3]	1.018	0.993	1.043	0.168
Sex	2.513	0.149	42.295	0.522
Hypertension	77.911	3.173	1913.238	0.008
Hyperlipidaemia	229858101	0.000		1.000
Diabetes mellitus	0.147	0.023	0.939	0.043
Lipid-lowering agents	17.989	0.664	487.240	0.086
Constant	0.000			0.073

AAA, abdominal aortic aneurysm; CI, confidence interval; Exp(B), odds ratio.

Supplemental Table 7 Patient demographics (at baseline) in extended prognostic study

Parameter		N (%)	Median (IQR)
Sex	Women	8 (12.1%)	
	Men	58 (87.9%)	
Age [years]			71.6 (11.0)
Body-mass index			27.7 (6.0)
Smoker status	Never	4 (6.1%)	
	Past	38 (57.6%)	
	Current	24 (36.4%)	
Nicotine pack-years			40 (35)
AAA family history	Yes	58 (87.9%)	
	No	8 (12.1%)	
Maximum AAA diameter [mm]			45.8 (12.4)
Aneurysm volume [cm³]			77.8 (67.0)
Maximum ILT thickness [mm]			12.3 (13.4)
Hypertension	Yes	57 (86.4%)	
	No	9 (13.6%)	
Hyperlipidaemia	Yes	54 (81.8%)	
	No	12 (18.2%)	
PAD	Yes	16 (24.2%)	
	No	50 (75.8%)	
Coronary heart disease	Yes	25 (37.9%)	
	No	41 (62.1%)	
Myocardial infarction	Yes	14 (21.2%)	
	No	51 (77.3%)	
	Unknown	1 (1.5%)	
Stroke	Yes	8 (12.1%)	
	No	58 (87.9%)	
Diabetes mellitus	Yes	17 (25.8%)	
	No	49 (74.2%)	
COPD	Yes	16 (24.2%)	
	No	50 (75.8%)	
Anti-platelet therapy	Yes	58 (87.9%)	
	No	8 (12.1%)	
Anti-hypertensive therapy	Yes	56 (84.8%)	
	No	10 (15.2%)	
Lipid-lowering agents	Yes	61 (92.4%)	
	No	5 (7.6%)	

AAA, abdominal aortic aneurysm; COPD, chronic obstructive pulmonary disease; ILT, intraluminal thrombus; IQR, interquartile range; PAD, peripheral artery occlusive disease.
